# Fundamentals for predicting transcriptional regulations from DNA sequence patterns

**DOI:** 10.1038/s10038-024-01256-3

**Published:** 2024-05-10

**Authors:** Masaru Koido, Kohei Tomizuka, Chikashi Terao

**Affiliations:** 1 Laboratory of Complex Trait Genomics, Department of Computational Biology and Medical Sciences, Graduate School of Frontier Sciences, The University of Tokyo, Tokyo, Japan; 2 Laboratory for Statistical and Translational Genetics, RIKEN Center for Integrative Medical Sciences, Yokohama, Japan; 3 Clinical Research Center, Shizuoka General Hospital, Shizuoka, Japan; 4 The Department of Applied Genetics, The School of Pharmaceutical Sciences, University of Shizuoka, Shizuoka, Japan

**Keywords:** Genome informatics, Genomics

## Abstract

Cell-type-specific regulatory elements, cataloged through extensive experiments and bioinformatics in large-scale consortiums, have enabled enrichment analyses of genetic associations that primarily utilize positional information of the regulatory elements. These analyses have identified cell types and pathways genetically associated with human complex traits. However, our understanding of detailed allelic effects on these elements’ activities and on-off states remains incomplete, hampering the interpretation of human genetic study results. This review introduces machine learning methods to learn sequence-dependent transcriptional regulation mechanisms from DNA sequences for predicting such allelic effects (not associations). We provide a concise history of machine-learning-based approaches, the requirements, and the key computational processes, focusing on primers in machine learning. Convolution and self-attention, pivotal in modern deep-learning models, are explained through geometrical interpretations using dot products. This facilitates understanding of the concept and why these have been used for machine learning for DNA sequences. These will inspire further research in this genetics and genomics field.

## Introduction

Large-scale consortiums have identified cell-type-specific regulatory elements through omics technologies. For example, in 2012, The Encyclopedia of DNA Elements (ENCODE) phase 2 conducted large-scale genomic assays for 147 different cell types and reported that 16.4% of the human genome is open chromatin regions (OCRs), and 4.2% of the human genome is likely to be enhancers that modulate transcription in a cell-type specific manner [1]. Seven years later, ENCODE phase 3 expanded the resources into >500 cell types and tissues and reported that 30.5% of the mappable human genome is open chromatin regions, and in total, 6.9% is candidate cis-regulatory elements (cCREs) with enhancer-like signatures [2]. Expanding publicly available resources and bioinformatics methods has contributed to numerous biological findings. In the research field of genome-wide association study (GWAS), these public genomic annotations have been widely used for the enrichment analysis of GWAS-identified variants and SNP heritability for human complex traits, providing a biological interpretation of their genetic architecture, such as the involvement of brain cell types in the genetic architecture of obesity (BMI) [3, 4]. Besides, genetic associations are highly enriched across various complex traits in the trait-specific non-coding regulatory regions, especially for active enhancer regions [3, 5, 6]. These publicly available resources combined with statistical enrichment analysis methods dissolve the genetic architecture of complex traits from GWAS results; however, detailed mechanisms behind genetic associations remain widely unclear due to our incomplete knowledge about allelic effects on transcriptional regulation.

To know genetic effects on transcriptional activities, there are three popular approaches: (i) molecular quantitative trait locus (QTL) study such as expression QTL (eQTL) [7–10], chromatin accessibility QTL (caQTL) [11–17], and histone mark QTL [18–20], (ii) experimental mutagenesis using reporter assay, including massively promoter reporter assay (MPRA) [21–24], and (iii) in silico mutagenesis using machine learning models trained on DNA sequences [25–29]. The concept, benefits, and limitations can be found in previous reviews such as [30, 31]. In this review, we first introduce in silico mutagenesis and then focus on providing its technical background for primers willing to understand transcriptional regulation using machine learning techniques.

### Overview of in silico mutagenesis

In silico mutagenesis is an approach to predict mutation effects from machine learning models trained to predict specific tasks by nucleic acid sequences (DNA or RNA). Tasks to predict from the input nucleic acid sequences are, for example, chromatin profiling, such as chromatin accessible regions and transcription factor (TF) binding sites [29, 32–34] and expression levels [25–28, 35], splicing [36], alternative polyadenylation [37], pathogenicity [38], and so on. The following four-step procedures are generally required for performing in silico mutagenesis. First, we design a machine learning model (or architecture) or choose it from the previous models (examples are shown later). Second, we prepare training, validation, and testing datasets. The training dataset is used for determining or updating weights by using pre-defined criteria. The validation dataset is used for selecting methods, hyperparameter tuning, and early stopping of the training procedure to avoid overfitting. The testing dataset is used to evaluate the final accuracy of the best model. Regarding input DNA sequences in training, reference genomes (such as hg19/GRCh37 and hg38/GRCh38) have been widely used instead of personal genomes. This strategy works well and is beneficial because whole genotype information for many omics datasets is often unavailable. Third, we train the machine learning models on the training dataset monitoring performance with the validation dataset, and evaluate the final performance in the testing dataset. The higher performance will ensure the validity of the following analyses. Fourth, we change the part of input DNA sequences into alternative alleles and calculate the changes in predicted values using the pre-trained model in step 3 (in silico mutagenesis). The alternative alleles are arbitrary; we can predict the mutation effects of alleles that have not been reported so far (or even do not exist in humans). To verify the performance of in silico mutagenesis, measuring the accuracy of in silico mutagenesis by comparing the in silico mutation effects with eQTL or MPRA results is essential. This evaluation will provide the threshold for a high-confidence list of mutation effects [27]. In the following sections, this review focuses on the first step, designing machine learning for genomics data.

### A brief history of machine learning models to predict regulatory elements from DNA sequences

The conventional machine learning approach uses k-mer, where we take k-continuous bases (if *k* = 5, for example, ACGCT) and count their frequency within input DNA sequences with a pre-defined length. The k-mer profile can be input features for many machine learning models. For example, in 2011, the kmer-SVM method took *k* = 3–10 within hundreds of DNA sequencing and used them as input features in support vector machine (SVM) where the predictability was mainly verified using EP300 binding sites from mice chromatin immunoprecipitation (ChIP)-seq experiments [39]. In their SVM framework, it was reported that, for input features, k-mer was better than the position weight matrix (traditional scoring matrix for TF motif) for known motifs [39, 40]. In 2014, gapped k-mer SVM (gkm-SVM) trained using ~316 bp input DNA sequences on human ENCODE ChIP-seq datasets was proposed [34], and in 2015, the high concordance of in silico mutation effects from gkm-SVM (deltaSVM) with the effect sizes from DNase I–sensitivity QTL, eQTL, and reporter assays including MPRA were shown [33].

Around 2015, convolutional neural networks (CNN) for genomics data showed higher predictive accuracy than the gkm-SVM [29, 32, 41]. For example, DeepSEA (deep learning-based sequence analyzer), a deep CNN model, predicts the on-off of regulatory elements within the center 200 bp in a 1 kb DNA sequence [32]. DeepSEA models trained on ENCODE and Roadmap ChIP-seq and DNase-seq datasets performed better than the gkm-SVM, even if customized gkm-SVM using the same length of input DNA sequences (1 kb) was used (Median area under the receiver operating characteristics curve (AUROC) of DeepSEA in their evaluation dataset was 0.958 while that of gkm-SVM was 0.896) [32]. This improvement indicates that the CNN-based approach is more suitable for incorporating long DNA sequences, enabling it to demonstrate higher accuracy than the gkm-SVM approach. After the success of DeepSEA, several CNN-based methods for predicting cell-type specific regulatory elements were also proposed. Basset is a CNN model that predicts peak on-off in the input 1,200 bp DNA sequences (centering on the midpoint of the peak) by training on DNase-seq peak Browser Extensible Data (BED) format files for 125 cell types from the ENCODE and 39 cell types from the Roadmap [29]. The mean AUROC of Basset in their evaluation dataset was 0.895, while that of gkm-SVM was 0.780 [29]. DeFine (Deep learning based Functional impact of non-coding variants evaluator) is a CNN model that predicts TF-DNA binding sites from 300 bp DNA sequences (centering ChIP-seq peaks) by training on TF ChIP-seq data of K562 and GM12878 cell lines [41]. DeFine authors reported the performance to classify regulatory non-coding variants from neutral variants in, for example, the HGMD (Human Gene Mutation Database) and the AUROC for Define-combine (combining scores using regression and classification versions) was 0.847, while that of DeepSEA was 0.822 and that of CADD was 0.727 [41]. From DeepSEA developers, advanced DeepSEA-derived architectures were proposed, such as DeepSEA beluga (increasing total window to 2 kb) [25] and deeper DeepSEA (doubling the number of convolutional layers) [42]. The improvement of deeper DeepSEA was marginal compared to the advancement of original DeepSEA over gkm-SVM: the average AUROC in their evaluation of deeper DeepSEA in this paper’s evaluation was 0.938, while that of original DeepSEA was 0.933 [42]. It is noted that different prediction tasks and strategies among those multiple models make it difficult to directly compare the other machine-learning models.

After 2018, methods to predict cell-type-specific gene expressions have emerged, where CNN is a crucial component for these approaches. Basenji, proposed by Basset developer, predicts read counts in every 128 bp bin in the input 131 kb DNA sequences using end-to-end CNN architecture by training on alignment files [26]. The mean Pearson correlation coefficient between predictions and measurements was overall 0.85 across 973 FANTOM5 CAGE dataset [26]. ExPecto, presented by DeepSEA developers, predicts expression levels in the center of the input DNA sequences with the 40 kb using CNN architecture (deepSEA beluga) and gradient boosting method [25]. The median Spearman correlation coefficient between predictions and measurements was 0.819 across the RNA-seq dataset of 218 tissues and cell types. Both methods (Basenji and ExPecto) were published in 2018 and were game-changers. Even after these developments, there have been improvements in the accuracy through many efforts, such as multi-task learning incorporating non-human datasets to increase accuracy for humans (Basenji2) [35], proper usage of long sequence information by partly replacing CNN architecture of Basenji2 by self-attention architecture (Enformer; a portmanteau of enhancer and transformer) [28], and predicting non-coding RNA expressions by improving model architecture of ExPecto and binary prediction (MENTR; mutation effect prediction on ncRNA transcription) [27]. Longer input DNA sequences have been used for predicting cell-type-specific gene expressions than those for predicting cell-type-specific accessibility and TF bindings. In ExPecto architecture, 40 kb sequences were used, and more than 40 kb sequences showed negligible performance gain [25]. On the other hand, MENTR, which specializes in non-coding RNA prediction, showed quantifiable improvement by using much longer input DNA sequences (200 kb) to achieve higher accuracy, especially for predicting enhancer RNA expressions [27]. The binary classification strategy in MENTR showed high capacity in predicting enhancer RNA expression levels [27] and, therefore, might make it easier to incorporate longer sequences’ information than the regression approach in ExPecto. Although Basenji2 architecture could not effectively use sequence information 20 kb from the TSS, partly replacing CNN by self-attention in Enformer increased it to 100 kb away [28]. The requirement of long DNA sequences for predicting expression levels indicates complex biology in transcription, such as 3D DNA contacts [28].

### CNN architecture and related essential techniques

As discussed, CNN is a fundamental machine-learning technique to predict cell type-specific regulatory elements and expression levels from long DNA sequences (>1 kb). CNN has been a representative method that made breakthroughs in the image recognition field [43–46]. Supposing the similarity of the data structure of images and DNA sequences will make it easier to understand why CNN succeeds in both areas. Images can be represented by height (*H*), width (*W*), and channel (*C*), where a black-white image has one channel (assuming that everything is white from beginning) and a color image has three channels (red, green, and blue). In general, CNN learns local information (neighbor pixels) of the picture in shallow layers [47] and then can recognize the parts of the image (e.g., ears, nose, etc., for human face recognition) in deeper layers, resulting in recognizing whole images from the combination of the parts in much deeper layers [48]. Similarly, the DNA sequences can be represented by length (*L*) and channel (*C*), where four channels (A, T, C, and G bases) are used, and local features (conventional motif sequence) are empirically important for predicting regulatory activities as motif analyses have done [49]. If integrating such local features determines the regulatory activities, CNN architecture will provide better performance for predicting them. Then, applying image recognition techniques is a reasonable strategy.

Geometric interpretation using the dot product (inner product) helps us understand why CNN can learn local features. The dot product is:

$${{{{{\boldsymbol{a}}}}}} \cdot {{{{{\boldsymbol{b}}}}}}={\sum }_{i=1}^{n}{a}_{i}{b}_{i}=\left|\left|a\right|\right|{{{{{\rm{|}}}}}}{{{{{\rm{|}}}}}}b{{{{{\rm{||}}}}}}\cos \theta$$

where ${{{{{\boldsymbol{a}}}}}}$ and ${{{{{\boldsymbol{b}}}}}}$ are size *n* vectors, and the angle between the two vectors is $\theta$. Depending on $\theta$, the dot product returns positive, zero, or negative values. From a geometric point of view, the dot product measures the similarity of two vectors: if the dot product has positive values, the two vectors are pointing in a similar direction ($\theta \, < \, {90}^{\circ} \;$); Especially when the two vectors are normalized, the larger the dot product is, the more similar the two vectors are. Based on this geometric property, we can interpret the convolution operation as searching a specific pattern (kernel) in the input data.

**Fig. 1:**
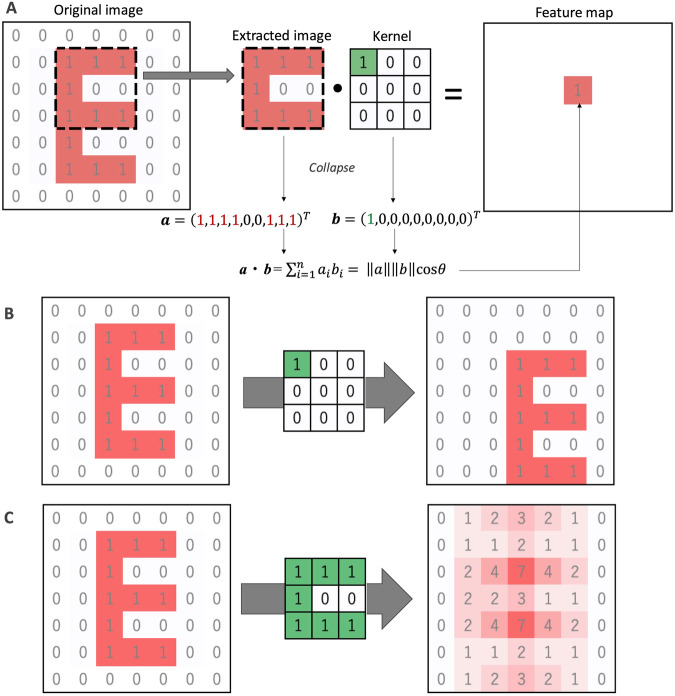
Conceptual representation of convolution operation. **A** Toy example of convolution operation using a 7 × 7 input image (original image) and 3×3 kernel. This image and kernel have two colors: 1 is red, and 0 is white in the input image, and 1 is green and 0 is white in the kernel. In the feature map, the output value for the location is highlighted in red. In this toy example, the bias parameter is not shown. **B**, **C** There are two examples of convolution using a kernel with one in the upper left and 0 in others (**B**) and a “C”-like kernel (**C**)

Suppose the input data is an image. Convolution operation calculates the sum of element-wise product between a small kernel and part of the input image (the same size as the kernel) over the input image (for a simple explanation, we ignore the bias parameter in this review), resulting in creating a feature map. This operation is geometrically equivalent to taking the dot product of the kernel over the input image to find a similar pattern in the input image (Fig. 1A). When we use a 3 × 3 kernel with 1 in the upper left and 0 in others, this kernel can find locations where “1” exists in the upper left, resulting in drawing a moving object in the feature map (Fig. 1B). When we use a 3 × 3 kernel with a “C”-like shape, this kernel can find a “C”-like shape in the input image, resulting in highlighting such region in the feature map (Fig. 1C). Similarly, if input data is a DNA sequence, it can be interpreted that convolution marks where the motif is located in the input sequence. Although, in these examples, we assume the fixed weights in the kernels, CNN learns weight parameters in the kernel via backpropagation. By preparing many types of trainable kernels, CNN can learn local features to find local objects.

In CNN, many techniques other than convolution operation are used. For example, max pooling (or average pooling) reduces verbose features in a small region and relatively increases windows for subsequent convolution. ReLU (Rectified Linear Unit), a function to calculate max values between an input value and zero, makes sparseness and adds non-linearity in the network [50]. Multi-task learning is helpful if we have many types of output values that are not independent [51]. Dropout is beneficial to avoid overfitting by preventing complex co-adaptations on training data (conspiracies) [52].

### Boosting

Some machine learning models, such as ExPecto and MENTR, use a two-step algorithm to predict transcription: the 1st step is CNN, and the 2nd step is a linear model (ExPecto) [25] and tree-based model (MENTR) [27] using a gradient boosting algorithm (not deep learning). Boosting is a popular ensemble learning method combining multiple prediction models. After the proposal of the boosting algorithm using weak learners in the 1990s [53, 54], Friedman reported gradient boosting in 1999, which performs sequential learning based on the gradient of any loss function [55]. Around 2014, Chen et al. developed and released eXtreme Gradient Boosting (XGBoost), an optimized and efficient version of gradient boosting [56]. ExPecto uses a linear model for a weak learner, and MENTR uses a decision tree [25, 27]. In general, tabular data-based machine learning for predictions tends to show better performance by tree-based approaches than deep learning-based approaches [57]. Researchers willing to develop new methods should consider better machine learning approaches based on the data types or compare the performance among several methods.

### Attention

The attention function returns weighted values based on the embeddings of the input vectors and their distance (i.e., similarity) [28]. In particular, a self-attention mechanism, “scaled dot-product attention,” is a popular attention function that uses three transformed vectors (keys, queries, and values) from the input sequence and calculates weighted values from attention weights from keys and queries (explained later). Transformer architecture relies entirely on the self-attention mechanism [58] and has become the gold standard in natural language processing (NLP). Soon after, Transformer architecture was also proposed for image recognition as a Vision Transformer [59]. Enformer is a model incorporating the self-attention mechanism in Basenji2 architecture to predict transcriptional regulation from DNA sequences, resulting in improved prediction accuracy, potentially through increasing long-range interactions in the input DNA sequences [28]. Although the name of Enformer looks like Transformer, Enformer architecture still relies on convolution layers.

**Fig. 2:**
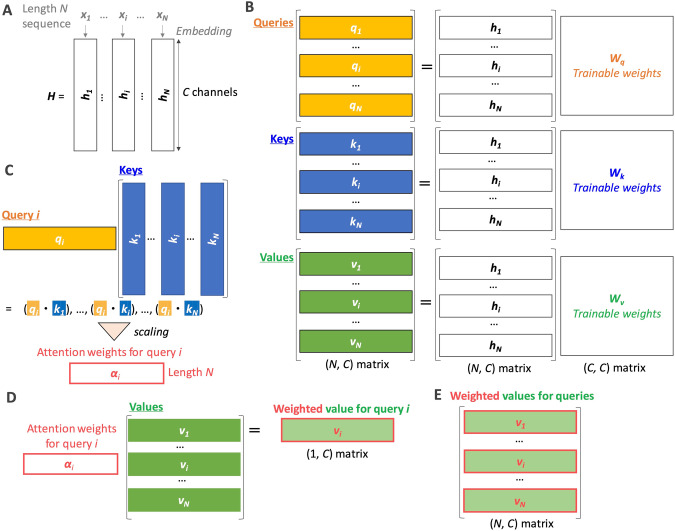
Conceptual representation of self-attention. **A** Embedding from original input sequences {*x*_*1*_ … *x*_*N*_} to {***h***_***1***_, …, ***h***_***N***_}. This embedding is trainable and required before the first self-attention procedure. **B** Preparing queries {***q***_***1***_, …, ***q***_***N***_}, keys {***k***_***1***_, …, ***k***_***N***_}, and values {***v***_***1***_, …, ***v***_***N***_} from the embedded vectors, where matrixes, ***W***_***q***_, ***W***_***k***_, and ***W***_***v***_, contain trainable parameters, respectively. The aligned two matrixes represent the matrix multiplication (the same after this). In the second and more self-attention procedures, the output from the previous self-attention procedure can be used instead of the shown embedded matrix. **C** Calculating attention weights for query *i* (***q***_***i***_). The aligned vector (on the left) and matrix (on the right) indicate the matrix multiplication (the same after this). The dot indicates the dot product. **D** Calculating the weighted value vector for the query *i* from the attention weights and values. **E** Weighted values for all queries (*i* = 1, …, *N*). The square brackets indicate that the collection of vectors (box) is treated as a matrix

The self-attention mechanism depends on dot product manipulation. As mentioned above, the dot product geometrically measures the similarity of two vectors. We assume a length *N* sequence (e.g., number of words in NLP). Each element can be represented by an embedded vector with a specific size (*C* channels). In Enformer architecture, the length *N* is 1,536, and the size *C* of the embedded vector is also 1,536, obtained after several convolution operations from length 197 K input DNA sequence [28]. In self-attention, we consider that there are length *N* input vectors {***h***_***1***_, …, ***h***_***N***_} (Fig. 2A) and transform them into key, query, and value vectors by trainable weight matrix with size [*C*, *C*], resulting in *N* vectors with length *C* for each item (keys, queries, and values) (Fig. 2B). Next, we select one query vector *i* (***q***_***i***_) and calculate the dot product with keys. The products are length *N* vectors representing the similarity between query *i* and each of the keys: {***k***_***1***,_ …, ***k***_***N***_}. After scaling, the products are called attention weights for query *i* (***α***_***i***_ = {*α*_*i,1*,_ …, *α*_*i,N*_}) (Fig. 2C). We can get weighted value vector *i* using these attention weights (Fig. 2D). Finally, for all *i* (*i* = 1, …, *N*), we can get *N* weighted value vectors (*i.e*., *N* × *C* matrix) (Fig. 2E). Because the size of the output matrix of self-attention is the same as that of the original embedded one (***H***, aligning {*h*_*1*_, …, *h*_*N*_}), we can do self-attention operations repeatedly. In the actual calculation, these operations are done using highly optimized matrix multiplication code and, therefore, the very first [58]. The trainable parameter is the three types of weight matrices for transforming into queries, keys, and values (each size: [*C*, *C*]) (Fig. 2B), as well as those for embedding (Fig. 2A). If we prepare multiple types of weight matrixes, we can aggregate them using additional trainable weights, called multi-head attention. Both Transformer and Enformer use eight attention heads.

As mentioned before, the attention weights can be determined by the similarity between queries and keys, and therefore, these weights are not trainable; this is the big difference from other types of machine learning approaches, including CNN, where such weights are directly trainable. Thanks to the flexible framework, self-attention can produce new embedded features depending on the similarity among input data. In the translation task in NLP, this characteristic enables one to guess the meaning from the context [60]. Similarly, Enformer was reported to learn to predict enhancer-promoter interactions without explicit their positional information [28].

One limitation of self-attention architecture is that the computational cost depends on the squared of the input sequence length, which will hamper the application in machine learning on DNA sequences because very long DNA sequences are required, especially for predicting cell-type specific gene expression levels, as discussed above. In fact, Enformer first shortened the input sequence from 197 kb to 1536 by many convolution layers and used self-attention. Overcoming this limitation may further improve current prediction accuracies.

## Discussion

This review briefly introduced the history and advancement of machine learning approaches to predict transcriptional regulation by DNA sequences alone. Although the powerfulness and usefulness of interpreting human genetic studies by in silico mutagenesis were proposed by several papers [25, 27], recent studies demonstrated that predicting personal transcriptome levels from personal DNA sequences is still challenging [61, 62], indicating more advanced machine learning models, strategy, or more large datasets will be required.
